# A cognitive multiplex network approach to investigate mental navigation and predict high-level cognition

**DOI:** 10.3758/s13428-025-02748-6

**Published:** 2025-08-25

**Authors:** Ofir Ganor, Gal Samuel, Massimo Stella, Yoed N. Kenett

**Affiliations:** 1 Faculty of Data and Decision Sciences, Technion – Israel Institute of Technology, Haifa, Israel; 2 CogNosco Lab, Department of Psychology and Cognitive Science, University of Trento, Trento, Italy

**Keywords:** High-level cognition, Mental navigation, Cognitive multiplex network, Creativity, Intelligence

## Abstract

High-level cognition, such as intelligence and creativity, are considered the hallmark of human cognition; however, their complexity hinders the identification of underlying common mechanisms. We focus on one such likely mechanism—mental navigation. We utilize converging computational methods to demonstrate how mental navigation—operationalized via verbal fluency tasks—predicts individual differences in creativity, intelligence, and openness to experience (the personality trait most closely related to them). Participants’ (*N* = 479) responses to two tasks—a 2-min animal fluency task and a 2-min generating synonyms of the word “hot” fluency task—were modeled over a multidimensional model (a cognitive multiplex network) of the mental lexicon. Quantitative measures of their mental navigation were used to build regression models that significantly predicted their assessed high-level cognition (replicating across both fluency tasks). Finally, we developed an online tool that capitalizes on our approach—the High-level Cognitive Prediction tool. Overall, we show how converging computational tools can elucidate the complexity of high-level cognition.

**Supplementary Information:**

The online version contains supplementary material available at 10.3758/s13428-025-02748-6.

## Introduction

High-level cognition is considered the hallmark of human cognition, and broadly refers to cognitive capacities of cognitive control, executive processes, working memory, planning, learning, intelligence, creativity, reasoning, and problem-solving (Yule et al., 2013). All of these capacities are biologically realized by the prefrontal cortex, which is uniquely developed in humans compared to other species (Diamond, 2013; Jung & Vartanian, 2018; O'Reilly, 2006). Yet, the complexity of these capacities is still far from understood, as these processes are typically context-dependent and evolve over time. Creativity, for example, is realized by the complex interactions of memory, attention, and cognitive control (Benedek & Fink, 2019; Benedek et al., 2023).

Rapid advancements in investigating high-level cognition are possible due to the development and application of computational modeling to elucidate such capacities (e.g., Lloyd-Cox et al., 2023). Here, we focus on the application of computational models to examine the role of mental navigation in high-level cognition, i.e., the ability to explore associated conceptual representations in memory (Todd & Hills, 2020). Such ability, also described as cognitive search, has received empirical interest in the past (Todd et al., 2012). This interest has largely focused on the degrees of freedom governing such search process—by models emphasizing two-variable clustering-switching or a single-variable random walk (Abbott et al., 2012, 2015; Hills et al., 2012; Hills et al., 2015; Jones et al., 2015). However, limited research has focused on the profile of the search itself (Hills, 2025; Hills & Pachur, 2012; Hills et al., 2013) or how it may facilitate individual variability in high-level cognition (e.g., Hart et al., 2022; Malaie et al., 2024; Ovando-Tellez, Benedek, et al., 2022). One prominent example of high cognitive capacity where mental navigation plays an important part is creativity.

Recent theories have highlighted the significance of cognitive mechanisms that facilitate mental exploration and exploitation that realize high-level cognitive capacities such as creativity (Beaty & Kenett, 2023) and general information-seeking behavior (Ivancovsky et al., 2024; Kenett et al., 2023). For example, extensive research has demonstrated how individuals with higher levels of creativity are more associative in their thinking, and can search more broadly and deeply in their memory (Beaty & Kenett, 2023; Benedek et al., 2023). However, like many other high-level cognitive capacities, creativity is difficult to measure and define (Green et al., 2024; Kaufman, 2019; Runco & Jaeger, 2012). Thus, here we flip the relation between creativity and associative thinking and ask the opposite question: Can the way a person searches their memory predict how creative they are? Or how intelligent they are?

To directly examine this question, we combine computational approaches with empirical assessments of high-level cognition. As a demonstration of the generality of our approach, we focus on creativity and intelligence, cognitive capacities that are difficult to measure and strongly related (Silvia, 2015). In addition, we examine the personality trait openness to experience, which is closely related to creativity (DeYoung et al., 2014; Kaufman et al., 2016; Oleynick et al., 2017). We computationally model how participants search their memory in a simple verbal fluency task and then apply regression methods to predict their high-level cognitive capacities. Surprisingly, there has been little research using computational methods to predict high-level cognition, and the bulk of this prior research is based on cognitive neuroimaging data (Pellegrini et al., 2018; Varoquaux & Thirion, 2014) or predicting cognitive decline (Ansart et al., 2021; Na, 2019).

Therefore, this study aims to predict scores for openness, creativity, and intelligence using simple behavioral tasks, specifically through a brief fluency task. We employ a computational cognitive multiplex network model (Stella et al., 2024) to assess these traits, moving beyond traditional self-report measures and standard behavioral assessments of verbal fluency tasks (Henderson et al., 2023). The success of our prediction model provides deeper insights into the role of mental navigation processes in high-level cognition and the relationship between personality and cognition, emphasizing the importance of semantic memory in understanding these traits. Critically, our aim here is to highlight the potential of applying cognitive multiplex network models in empirical psychological research, focusing on high-level cognition as a specific case study. To facilitate the practical application of our findings, and as a proof of concept of the general potential of such a model, we developed a graphical user interface (GUI) called High-Level Cognitive Prediction (HiCoP). This tool allows users to complete the fluency task and receive predicted scores for openness, creativity, and intelligence, making our research accessible and engaging for a broader audience.

## High-level cognitive capacities

In this study, we focus on high-level cognitive capacities that have been shown to relate to semantic memory—the cognitive system that stores facts and knowledge (Kumar, 2021)—such as creativity, intelligence, and the personality trait openness to experience. Semantic memory represents a key component of the so-called mental lexicon (Stella et al., 2024), and plays a critical role in high-level cognition such as creativity and intelligence (Gerver et al., 2023; Kenett, 2025; Li et al., 2024). Specifically, the current study quantitatively examines mental navigation processes that operate over the mental lexicon, by operationalizing mental navigation as a search process over conceptual representations within it (Benigni et al., 2021; Hills & Kenett, 2022; Todd & Hills, 2020). While high-level cognitive capacities involve multiple cognitive processes (Benedek & Fink, 2019), semantic memory has a general role in realizing them, as well as the processes that operate over semantic memory such as semantic priming or associative thinking (Abraham & Bubic, 2015; Collins & Loftus, 1975; Fradkin & Eldar, 2023).

*Creativity* is defined as the generation of novel and useful ideas (Green et al., 2024; Runco & Jaeger, 2012), related to a search process that “moves away"from common ideas executive processes that guide such searches (Beaty & Kenett, 2023; Beaty et al., 2014; Benedek & Neubauer, 2013; Kenett, 2023, 2025; Mednick, 1962; Volle, 2018). The merging of remote, weakly related concepts into a novel and applicable concept defines the creative process, as described by the associative theory of creativity (Beaty & Kenett, 2023; Mednick, 1962). Thus, creativity involves search processes that are impacted by ones’ semantic memory structure (Hills & Kenett, 2025; Kenett, 2025). Importantly, multiple studies have shown how variation in semantic memory structure relates to individual differences in creativity assessed as lab-based measures of creative thinking (Benedek et al., 2017; Kenett et al., 2014) or self-reported measures of creative potential (Kenett et al., 2016; Ovando-Tellez, Kenett, et al., 2022). Such pioneering research findings are slowly starting to translate into cognitive-inspired online applications that utilize such research (Wise & Kenett, 2024). Machine learning (ML) methods were used in prior neuroimaging studies to predict the optimal whole-brain functional connectivity that predicts creativity (Beaty, Kenett, et al., 2018; Frith et al., 2021; Ovando-Tellez, Kenett, et al., 2022).

*Intelligence* is considered the hallmark of human capacities; it is critical in multiple real-life applications and has garnered considerable attention in psychological and cognitive research (Kent, 2017). It encompasses problem-solving abilities, logical reasoning, and the capacity to acquire and apply knowledge. An individual's intelligence can impact their health, from physical fitness and obesity to alcoholism and infant mortality (Gottfredson & Deary, 2004). One facet of human intelligence is fluid intelligence (*Gf*). Kenett et al. (2016) have shown how higher *Gf* relates to a more structured semantic memory network. More recently, Li et al. (2024) showed how crystallized intelligence (*Gc*)—related to ones’ vocabulary knowledge—relates to variation in semantic memory structure. ML methods were used in prior studies to predict fluid intelligence. These works mainly come from neuroscience, using magnetic resonance imaging (Saha et al., 2021; Tamez-Pena et al., 2019; Wen et al., 2022) and cortical measurements (Zhu et al., 2018) paradigms.

*Openness to experience* is closely related to curiosity, creativity, a drive to learn new things, and having diverse hobbies. Among the Big-5 personality traits, openness to experience is the personality trait that is most strongly related to cognitive capacities (Zillig et al., 2002), including intelligence, working memory, semantic memory, and creativity (Christensen, Kenett et al., 2018; DeYoung et al., 2012; Kaufman et al., 2016). This personality trait is complex in nature, being related to different aspects of high-level cognition, specifically creativity and intelligence (Ashton et al., 2000; Christensen, Cotter, et al., 2019; Christensen, Kenett, et al., 2018). Christensen, Kenett, et al. (2018) have shown how variation in openness scores relates to variations in semantic memory structure, variations that are similarly exhibited in relation to individual differences in creativity.

## Cognitive networks—from single layers to multiplex models

The advancement in studying such high-level cognitive capacities, and especially the role of semantic memory and the processes that operate over it in these capacities, is made possible by recent computational advances. One approach that has been gaining popularity is cognitive network science (Siew et al., 2019). A cognitive network is a representation of associative knowledge where network nodes represent concepts and network links indicate associations or relationships between nodes. Cognitive network science applies computational network science methodologies that are based on mathematical graph theory. These computational tools have been applied to study broad cognitive domains such as language, memory, learning, aging, and creativity (Hills, 2025; Kenett, 2025; Kenett & Hills, 2022; Siew et al., 2019).

However, most current cognitive network science research studies networks encompassing only one type of link, such as semantic memory networks of free associations or phonological networks of sound similarities. Yet conceptual representations are complex in terms of their multidimensionality, i.e., they include different levels/layers that reflect different types of features (phonology, semantics, etc.). Thus, studying multidimensional—or multilayer—cognitive networks is needed to advance our understanding of the complexity of the human mind (Hills & Kenett, 2022; Stella et al., 2024).

Current research is exploring a model of a cognitive multiplex network — a complex multidimensional cognitive structure, that can be operationalized as a multilayer network model comprising different networks, one per layer (Stella et al., 2024). Such a cognitive multiplex network model includes different cognitive dimensions, such as phonology and semantics, and has been demonstrated as a powerful approach for studying various cognitive phenomena in typical and clinical populations (see Stella et al., 2024, for a recent review).

**Fig. 1:**
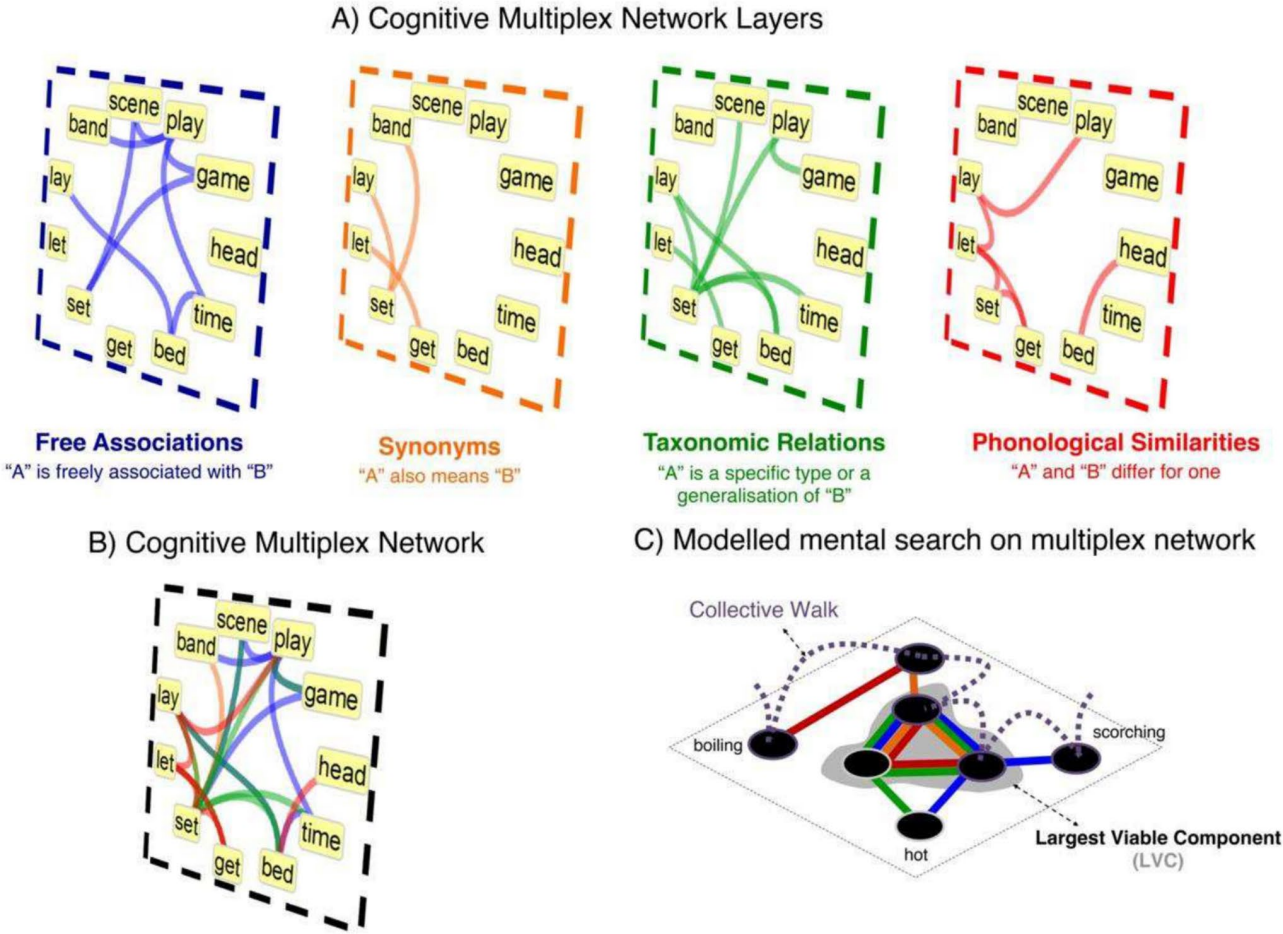
Multiplex network and the largest viable cluster. **A** In this cognitive multiplex network structure, nodes represent concepts replicated across four layers, namely free associations, synonyms, taxonomic relations, and phonological similarities. **B** All layers can be condensed into one edge-colored network, where links of multiple colors coexist. Each color represents one layer (e.g., red for phonological similarities). **C** In these edge-colored networks, we can model the fluency data as a walk including multiple nodes from the multiplex network. This walk can also include nodes inside the largest viable cluster (LVC), i.e., the largest set of nodes that are simultaneously connected across all layers. Measuring network features of these walks corresponds to measuring novel aspects of recalls from a fluency list

The cognitive multiplex network preserves the links from all independent layers and merges the independent layers into one multiplex network (Fig. 1; Stella et al., 2024). Typically, the layers include lexical information consisting of a synonym layer, a phonological layer, an associative layer, and a hypernym/hyponym layer, which have been shown to provide different (nonoverlapping) sets of links among the same set of concepts across layers (Stella et al., 2018). Combining these multiple layers reveals a component called the largest viable cluster (LVC). The LVC is a component of the multiplex network that consists of the largest collection of nodes which are connected among one another simultaneously across all multiplex layers (Fig. 1; Stella et al., 2018). Past studies in a cognitive multiplex network with 8,000 English concepts showed that the LVC is composed of highly concrete, familiar words, and its emergence is tied to explosive stages of language acquisition (Stella et al., 2018).

A few studies have shown how the cognitive multiplex network, and the LVC in particular, can be utilized to investigate high-level cognition. Stella and Kenett (2019) demonstrated its ability to classify individuals with lower and higher levels of creativity based on a simple semantic fluency task. The authors operationalized how these individuals"walked"on the multiplex network based on their responses in a semantic fluency task (where they listed names of animals). The authors then used an ML approach to classify participants, based on various measures computed on the cognitive multiplex network, into lower- or higher-creativity categories, with high accuracy rates (Stella & Kenett, 2019). In line with classical theories on creativity (Kenett, 2025), Stella and Kenett (2019) found that individuals with higher creativity tended to generate animal names outside the LVC, exploring more of the periphery of the cognitive multiplex network model.

Similar to the findings of Stella and Kenett (2019), Samuel et al. (2023) demonstrated that it is feasible to predict openness to experience scores by utilizing cognitive multiplex networks combined with ML models. Similarly to creativity, classifying participants as more open, as well as predicting general individual differences in openness scores, was related to mental navigation primarily in the periphery and less in the LVC of the cognitive multiplex network model (Samuel et al., 2023).

## The current study

The studies by Stella and Kenett (2019) and Samuel et al. (2023) primarily served as proof-of-concept investigations, exploring the effectiveness of a cognitive multiplex network in predicting complex behaviors. In the current study, we aim to highlight and demonstrate the significance of utilizing a cognitive multiplex network model in empirical cognitive psychological research. To achieve this, we aim to replicate and extend these previous works by using measurements from a cognitive multiplex network and regression methods to predict various aspects of complex high-level cognition, all based on a single fluency task that takes 2 min. Furthermore, we aim to generalize our findings by examining two different categories in the fluency task—animals and synonyms of “hot”—to highlight the general role of mental navigation in high-level cognition.

**Fig. 2:**
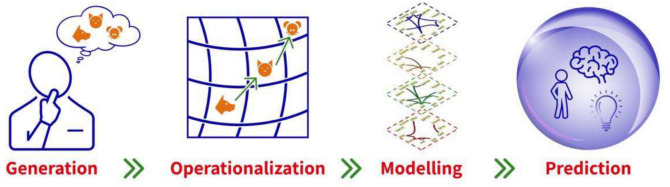
Illustration of study design. Participants’ responses *generated* in a verbal fluency task are used as an *operationalization* of their mental navigation over their memory. Theis behavior is *modeled* as a search process over the cognitive multiplex network model, and features of participants’ search over the multiplex are computed. These features are then used to train ML models to predict high-level cognitive capacities, such as creativity, openness, and intelligence

The novelty of this study lies in its attempt to predict the value of a cognitive capacity using only a single, simple behavioral task (see Fig. 2 for an illustration of the design of our study). We do so by collecting data from a large sample of participants, including measurements of verbal fluency, creativity, intelligence, and personality traits. This sample (*N* = 479) is considerably larger than those in the previous studies of Stella and Kenett (2019; *N* = 94) and Samuel et al., (2023; *N* = 163), allowing us to achieve ample power for reliable prediction by our trained ML model.

The tasks used in the current study to compute cognitive multiplex network measures of mental navigation are semantic fluency tasks, which consist of an operationalization of participants’ mental navigation over memory (Benigni et al., 2021). In a fluency task, participants are required to generate as many category members as possible in 2 min. This task was previously shown to be useful when examining how people search through their memory (Abbott et al., 2015; Hills et al., 2012; Hills et al., 2015; Ovando-Tellez, Benedek et al., 2022), and allows us to trace the steps of participants, navigating their mental path on the lexical structure. To replicate and generalize our findings, we analyzed performance in two different fluency tasks—generating animal names or generating synonyms to the word “hot”. Both categories have been used in creativity research (Beaty, Kenett, et al., 2018; Kenett et al., 2016), and in our previous work with the cognitive multiplex network model (Samuel, Stella et al., 2023; Stella & Kenett, 2019).

We aim to predict individuals’ openness to experience, creativity scores encompassing both creative thinking and creative potential, and intelligence based solely on participants’ mental navigation, without any additional self-assessments or other tests on the participants. This is conducted via an ML model, specifically a supervised learning model tested through a leave-one-out cross-validation approach (Alpaydin, 2020). Our supervised approach unpacks fluency responses as a collective walk on the cognitive multiplex network, whose structural measures are calculated based on each participant’s performance in the semantic fluency task. As such, we move away from standard analysis approaches of semantic fluency (Henderson et al., 2023) and focus on the process of the search itself rather on the retrieved content or number of responses, by computing several features that quantify participants’ mental navigation over the cognitive multiplex network.

Using these calculated features, we train ML models for each of the four traits we aim to predict. The use of supervised learning models for predictions is a widely popular approach. For example, it is used to predict hospitalization due to heart diseases (Dai et al., 2015), and slope stability (Lin et al., 2018). However, to the best of our knowledge, little to no cognitive research has been conducted with similar ML approaches. Specifically, we expect to be able to replicate the differences in how participants with different levels of creativity, openness, and intelligence rely on the LVC in retrieving category-member words in a semantic fluency task (Samuel et al., 2023; Stella & Kenett, 2019).

In the learning process, we implemented two different methods to identify the most significant features for predicting a target variable within a dataset. Feature selection was essential to reduce dimensionality, avoid overfitting, and improve model interpretability by focusing on the most informative features. We chose these specific methods because each captures unique types of relationships: a correlation-based method (Hall, 1999) to capture linear associations, and a boosting method, CatBoost (Hancock & Khoshgoftaar, 2020), to capture more complex, nonlinear relationships. These methods helped us rank features from highest to lowest importance for each target dependent variable (e.g., creative potential), allowing for systematic experimentation with different feature sets by adding features incrementally to train a linear regression model (Samuel et al., 2023).

Additionally, we modeled each of the four target dependent variables (DV) separately, given their independence. For each target DV, we experimented with the two feature selection methods to determine which method better captured the data structure for optimal prediction performance. This approach allows us to identify the most suitable features and selection technique tailored to each target variable's unique characteristics. Before training, we scale the features to ensure they are normalized, which helps improve the model's performance, and use L2 regularization. To ensure robust evaluation, the model is trained using leave-one-out cross-validation (Alpaydin, 2020), where it is trained on (*n* − 1) samples and tested on the remaining single sample, with *n* being the total number of samples. The mean squared error (*MSE*) between the predicted and actual test values is calculated to assess the model's performance. The feature whose removal results in the smallest increase in prediction error is deemed the least important and is permanently excluded from the model. This iterative process continues until the optimal set of features is identified, thus minimizing prediction error. We save the best model for each target DV, along with the scaler used for normalization, so that the trained model can be applied to new data. Through this process, we refine the feature set, train the regression model, and evaluate its performance—all fundamental aspects of ML aimed at enhancing predictive accuracy.

Finally, for the best-performing models we identify, we developed a GUI called HiCoP (High-Level Cognitive Prediction). This interface serves as a proof of concept for the potential of applying cognitive multiplex network modeling in empirical research, and allows users to perform the fluency task and obtain predicted scores for these four traits (https://tinyurl.com/y4kszemt).

In line with our previous studies (Samuel et al., 2023; Stella & Kenett, 2019), we expect to build ML models that predict individual differences across our four DVs, based on multiplex features computed over participants’ performance in the fluency tasks. Specifically, we hypothesize that people who are more creative and open will be characterized by mental navigation processes that are less reliant on the LVC. In addition, based on the strong relation between creativity and intelligence (Silvia, 2015), we expect to find similar characteristics related to individual differences in intelligence.

## Methods

### Participants

Sample size was selected based on previous work showing how ML regression methods are sensitive to sample size when aiming to predict individual differences in cognitive capacities (Cui & Gong, 2018), highlighting the need for a large sample size.

In total, 500 individuals who were recruited on Prolific Academic participated in the study, for which they were compensated £8. Twenty-one participants who did not comply with task instructions (e.g., did not generate animal names in the animal fluency task) were excluded from the final analysis. Thus, the final analyzed sample consisted of 479 participants (242 female, five participants did not specify; mean age = 37.15 years, *SD* = 11.23 years; mean years of education = 15.61 years, *SD* = 4.66 years) from predominantly English-speaking countries recruited from the Prolific Academic participant pool. They were compensated accordingly and signed a consent form. The study was approved by the Technion – Israel Institute of Technology Institutional Review Board.

## Materials

### Semantic fluency

According to standard procedure (Ardila et al., 2006), participants had 2 min to generate as many animal category members (i.e., types of animals) as they could think of. In addition, based on Samuel et al. (2023), participants generated synonyms of the word *hot* for 2 min. Analysis of the *hot* synonym data is reported in the supplementary information (SI).

### Creativity assessments

#### Creative potential

Creative potential was assessed via a self-report questionnaire, the Inventory of Creative Achievements and Activities (ICAA; Diedrich et al., 2018). ICAA is a commonly used for assessing individual differences in real-life creativity. It measures the frequency of engagement in everyday creative activities and creative achievements in eight different domains (e.g., arts, writing, cooking; Diedrich et al., 2018). For each participant, numeric responses from all domains are summed to compute a general creativity achievement and activities score (Ovando-Tellez, Kenett, et al., 2022).

#### Creative thinking

Creative thinking was assessed via the widely used in-lab Alternative Uses Task (AUT). In this task, participants are presented with an object and are then required to think of as many uses for the object as they can (Acar & Runco, 2019). It consisted of three items—broom, belt, and pencil—and each participant performed the task for all objects in randomized order. Response scores were computed with an automated scoring method (Organisciak et al., 2023) that uses a large language model, which was trained on a very large dataset of human scores of AUT responses and shows great accuracy in scoring new responses. We then took the maximum score response for each object for each participant and averaged the scores for the three objects to obtain each participant’s AUT score.

### Personality traits

The Big-5 personality traits, including openness (and corresponding facets), were assessed using the 240-item NEO-PI-3 questionnaire (McCrae et al., 2005). To assess openness, we follow the method conducted by Samuel et al. (2023). Of the NEO-PI-3 items that measure openness, we performed confirmatory factor analysis via the weighted least squares mean and variance-adjusted (WLSMV) estimator to estimate factor scores. This analysis was conducted using the *lavaan* package in R (version 0.6.10; Rosseel, 2012); the factor scores were used for subsequent prediction analyses.

### Intelligence assessment

Participants completed two intelligence tasks commonly used in past work on intelligence and creativity (Beaty & Silvia, 2012; Frith et al., 2021; Kenett et al., 2016). The tasks assessed fluid intelligence (*Gf*) and included (1) a number series task (15 items, 5 min), which presents sequences of numbers that change based on a rule and asks participants to select the next sequence (Thurstone, 1938), and (2) a series completion task from the Culture Fair Intelligence Test (13 items, 3 min), which presents sequences of three changing images (small line drawings) and asks participants to select the next image that fits the rule governing their change (Cattell & Cattell, 1961/2008). Finally, participants’ intelligence scores were computed as the average amount of correct responses from the two tasks.

### Cognitive multiplex network analysis

#### Cognitive multiplex network construction

 Our multiplex network consisted of four systems, all including the same set of 16,000 English words but linked across four layers: free associations, synonyms, phonological similarities, and hypernyms/hyponyms. To create the cognitive multiplex network, all the layers were treated as undirected. Data for all layers except for free associations were obtained from the WordData repository (Wolfram Research, Champaign, IL, USA), available through the Mathematica 11.3 program. The WordData dataset is based on WordNet 3.0 (Miller, 1995), which is a dictionary that includes information about word–word similarities as computed from English dictionaries (Stella et al., 2018). Specifically, the multiplex includes the following four layers:

- Free association layer: created using data on associations elicited by participants from the Small World of Words project (De Deyne et al., 2019). Only links that were elicited more than 10 times were considered eligible, so that the association layer featured the same link density as the other multiplex layers.
- Synonym layer: consists of word–word relations that represent meaning overlapping between the words, such as *hot* and *warm*.
- Phonological layer: consists of word–word relations that represent a one-phoneme difference between words, such as *cat* (kæt) and *bat* (bæt).
- Hypernym/hyponym layer: consists of word–word relations that represent generalization and specification, such as *bird* and *eagle*.

#### Multiplex measures

After creating the multiplex, we computed the largest viable cluster (LVC; see Baxter et al., 2016), which is the largest cluster of words that are connected across all layers. Similar to Stella and Kenett (2019), we computed several cognitive multiplex network measures and used multivariate statistical analyses to determine which variables significantly differed across the groups.

We used the fluency tasks responses of each participant to identify where the participant “walked” on the network, once with the “hot” synonyms task and once with the animal names task. Then we computed multiple measures for each participant (Stella & Kenett, 2019). 

The measures focus on aspects such as the interaction of each participant with the LVC, the entropy of paths participants used in their mental navigation, and the number of responses each participant generated in general and within and outside of the LVC (see Table 1).

**Table 1:** List of multiplex network properties assessed from participants fluency lists

Name	Definition
Number of responses	Number of responses in a fluency list
Coverage per response	Average number of nodes visited in the multiplex shortest paths connecting any two consecutive responses in a fluency list. This is a measure of coverage because it identifies how many nodes in the multiplex network should be covered to transition from one response to the next. The average is performed over the number of consecutive pairs of responses
Fraction of responses in LVC	Fraction of words in the fluency list being part of the LVC
Fraction of LVC accesses	In the collective walk, check how many nodes in the shortest paths, including endpoints, belong to the LVC. Endpoints are considered only once, avoiding repetitions. This number is then divided by the total number of nodes visited in the collective walk
Entropy of LVC accesses	In the collective walk, check whether one node is (1) within the LVC but (2) preceded by another node outside of the LVC. When these two conditions apply, substitute the node in the LVC with a 1. After having iterated this for all nodes in a collective walk, substitute all other non-1 nodes with 0. On the resulting binary list, compute the Shannon entropy. This counts the entropy of entries of the collective walk within the LVC
Entropy of LVC overage	In the collective walk, replace any node in every shortest path, including endpoints, with a 0 if the node is outside of the LVC, or 1 otherwise. Endpoints are considered only once, avoiding repetitions. On the resulting binary list, obtained with the above replacements, compute the Shannon entropy. This counts the entropy of words in the LVC met during the collective walk
Entropy of LVC responses	In the fluency list, replace any response with a 1 if it belongs to the LVC or with a 0 otherwise. On the resulting binary list, compute the Shannon entropy. This counts the entropy of responses in the LVC in a fluency list
Maximum permanence in LVC	In the collective walk, find the maximum number of consecutive nodes visited where all nodes are in the LVC
Median permanence in LVC	In the collective walk, find the median number of consecutive nodes visited where all nodes are in the LVC
Max out	In the collective walk, find the maximum number of nodes visited where all nodes are outside of the LVC
Median out	In the collective walk, find the median number of nodes visited where all nodes are outside of the LVC
Distance from *hot* per response	For every response, measure its shortest path length to the category identifier *hot*. Divide the sum of the lengths by the number of responses
Accesses to LVC from *hot*	Average number of visited nodes in the LVC in all multiplex shortest paths between one response and the category identifier *hot*
Start in the LVC	Flag for the first response in a fluency list being (or not) in the LVC
Fraction of typos	Percentage of incorrectly spelled responses
Norm 1	A normalized version of the maximum permanence in the LVC, divided by the number of responses
Norm 2	This is a normalized version of the average permanence in the LVC, divided by the number of responses

Table 1 can be better understood by formalizing an observed fluency list as an ordered set ${L}_{j}={({r}_{i}^{\left(j\right)})}_{i}$ of responses ${r}_{i}^{\left(j\right)}$ provided by a given participant *j*. For instance, the example fluency list ${L}_{1}=\mathit{\{}dog{,}\,cat{,}\,lion\}$ would be relative to participant $j=1$ and include $L=3$ responses, namely ${r}_{1}^{(1)}=\mathit{dog}$, ${r}_{2}^{(1)}=\mathit{cat}$, and ${r}_{3}^{(1)}=\mathit{lion}$. Given the observed responses, one could assume that (1) the produced concepts are the endpoints of a mental navigation process and that (2) such a process exploits the shortest paths on a multiplex network representation of the mental lexicon (Stella et al., 2024). By following these two assumptions, one can introduce the notion of a collective walk, ${w}^{(j)}=({s}_{\text{1,2}}^{(j)},{s}_{\text{2,3}}^{(j)},\dots ,{s}_{L-1,L}^{(j)})$ as the sequence of shortest paths ${s}_{i,i+1}^{(j)}$ taking place on a given multiplex network and connecting consecutive responses ${r}_{i}^{(j)}$ and ${r}_{i+1}^{(j)}$ in the fluency list produced by participant* j*. In our example, the collective walk could be

1 $${w}^{(1)}=\left(\left(do{g}{{,}\,hom{e},}\,ca{t}\right),\left(ca{t}{,}\,felin{e}{,}\,lio{n}\right)\right).$$

Another example of collective walk is visualized in Fig. 1C for the synonyms of “hot”. As is evident from the network visualization, a collective walk contains nodes (words) that were not mentioned by a given participant. Instead, these are concepts mediating the shortest paths between responses observed in a fluency list. Collective walks are meant to represent sequences of nodes linking responses on a multiplex network. Indeed, collective walks are one way of modeling mental search—they have been tested successfully in previous works related to modeling creativity (Stella & Kenett, 2019) and personality traits (Samuel et al., 2023)—among many other possibilities in the relevant literature (cf. Hills, 2025). The advantage of reconstructing collective walks (via shortest paths on cognitive multiplex networks) is the possibility of operationalizing fluency lists by counting operations on a multiplex structure, for example, counting how many nodes are covered in shortest paths, counting how many shortest paths go through a given set of nodes, and measuring network distances, among many others. In the example ${w}^{(1)}$, only *cat*, *dog*, and *home* belong to the largest viable component of the multiplex network, while *feline* and *lion* are outside it. Similarly, one could count the probability of a node being in the LVC during a collective walk and thus endow walks with entropy measures. Table 1 summarizes the measures extracted from collective walks here and used in our ML pipeline.

### Machine learning analysis

#### Data splitting

The data-splitting method we used was leave-one-out cross-validation (LOOCV; Alpaydin, 2020), in which all data points except one are used to train the model, and then the trained model is tested on the remaining data point. In this method, the split reoccurs multiple times so that each data point (i.e., the vector of measures relative to individual participants) is used as the test set exactly once. We use LOOCV to account for the sample size of this study while maximizing size and variability in the test set, thus reducing overfitting and producing more robust ML models (Alpaydin, 2020).

#### Feature selection

Feature selection techniques play an important role in reducing the training time and decreasing the number of features from the original set by choosing only informative and relevant features and eliminating redundant ones to increase classifier and model performance (Al-Sarem et al., 2020). Feature selection methods are often used for predictive models that are based on high-dimensional data. However, high dimensionality tends to result in information redundancy. To reduce the risk of overfitting, feature selection can be performed before constructing a model in order to extract the most important and useful information from the original dataset (Luo et al., 2021).

In our feature selection process, we employed two different methods to ensure robust selection of the most relevant features for our model. The use of multiple feature selection techniques provides a comprehensive view of feature relevance from different perspectives (Visalakshi & Radha, 2014). Correlation-based feature selection (CFS) offers a statistical approach by evaluating the direct correlation between each feature and the target variable, helping to identify features that have a strong linear relationship with the target (Hall, 1999). In contrast, CatBoost (Hancock & Khoshgoftaar, 2020) feature selection leverages tree-based models to rank features by their importance in improving model performance, capturing complex interactions and nonlinear relationships.

#### Correlation-based feature selection (CFS)

This method evaluates each feature's correlation with the target variable and selects the most relevant ones based on Pearson correlation (Hall, 1999). The central hypothesis is that good feature sets contain features that are highly correlated with the class (Hall, 1999).

#### CatBoost feature selection

CatBoost ranks features by their importance within a gradient boosting framework (Hancock & Khoshgoftaar, 2020). It was employed for feature selection by leveraging its tree-based learning process to compute feature importance. The model calculates importance scores for each feature, reflecting their contribution to minimizing prediction error, which was measured using the root mean square error (*RMSE*) in our case. Features were ranked in descending order of importance, enabling systematic identification of the most influential features in the dataset. Furthermore, CatBoost optimizes feature splits through its advanced gradient boosting algorithm, ensuring precise and efficient division of data across decision trees to enhance model performance (Hancock & Khoshgoftaar, 2020).

Using these two methods, separate lists of key features were created in descending order, with the most influential features at the top. Each method identifies the features most relevant to the target variable, reflecting different behaviors of the data: CFS prioritizes based on correlation, while CatBoost relies on tree-based importance metrics.

#### Models

 To predict a target score, a learning model is needed. Linear regression was chosen for the prediction, as it allowed us to easily compare goodness of fit between models, using Pearson's *R* and mean squared error (*MSE*). In the training phase, the StandardScaler method was applied to the features to normalize the data, setting each feature to have a mean of 0 and a standard deviation of 1. This scaling standardizes the feature ranges, enhancing model performance and speeding up convergence. Normalized features also ensure that no single feature disproportionately influences the model. Both a standard linear model and a regularized version with L2 regularization were used. L2 regularization, also known as ridge regression, is a technique that adds a penalty equal to the square of the magnitude of the coefficients to the loss function, helping to prevent overfitting by discouraging large weights in the model.

We experimented with different numbers of features by starting with the most relevant feature and incrementally adding the next in order of importance, observing the impact on model performance at each step. To select the best model, *MSE*, *R*, and *p*-values were compared. For new predictions, the same scaler from the training data was applied to the input data for consistency, ensuring that the new data aligned with the scale of the trained model. Finally, predictions were normalized to a 0-to-1 scale for clarity and comparability across each target.

## Statistical analysis

The initial phase of our analysis involved calculating multiple multiplex measures for each participant based on their fluency response lists. Pearson’s correlation tests were then conducted to examine the relationships between each multiplex measure and the dependent variables creative potential, creative thinking, openness, and intelligence. Using the best models identified, these models could subsequently be integrated into our GUI for practical application.

## Single-layer null models

The LVC is a genuine multiplex element, since it emerges from the combination of the topologies of each layer. Nodes in the LVC share the feature of being mutually connected across each multiplex layer at the same time. This multiplex nature also means that the single-layer counterpart of an LVC is a set of nodes that are mutually connected on that single layer, i.e., the single-layer counterpart of an LVC would be the largest connected component—or LCC—in that layer.

To compare the predictive performance of the LVC against single-layer null models, we test how restricting connectivity to a single layer only, rather than the whole multiplex network, and using that layer’s largest connected component rather than the LVC both impact the performance of the same regression models tested for the multiplex/LVC case. Note that in these single-layer models we use the same sets of features selected in the LVC model, thus enabling direct comparisons between models. Through these comparisons we can quantify whether the LVC, emerging from the combination of multiple dimensions of the mental lexicon, leads to better predictions compared to any individual aspect, such as considering only free associations or phonological similarities. We carry out these comparisons in terms of Pearson’s correlations between predictions and empirical scores.

## Procedure

All participants completed an approximately hour-long online questionnaire on Qualtrics, which included four attention checks. Participants were asked to endorse a consent form to indicate they agreed to participate in the study. Upon consent, participants received an explanation of the course of the experiment. All participants completed all tasks. Finally, they filled out a demographic questionnaire. The order of the tasks was randomized across participants.

## The HiCoP web application

The HiCoP tool is an interactive web-based application developed using oTree, an open-source platform for creating experimental social science studies (Chen et al., 2016). The tool is hosted on Heroku, a cloud platform that simplifies deployment and scaling of web applications (Danielsson et al., 2021). For calculating the multiplex features, we utilized the NetworkX library in Python, a powerful tool for the creation, manipulation, and study of complex networks (Platt, 2019). Specifically, we follow the same approach taken by Nurmi et al. (2024), which discusses the development and capabilities of the pymnet library for multilayer network analysis. NetworkX provided essential data structures and tools for creating, manipulating, and studying complex networks, efficiently handling multiplex structures as was described in Nurmi et al. (2024). This combination of technologies allows HiCoP to efficiently perform real-time calculations and deliver trait scores to users.

### HiCoP tutorial 

Our online tool provides an easy-to-use interface for assessing mental navigation through a brief fluency task. In the following, we outline the complete process, from accessing the tool to interpreting the results.

1. Accessing the tool: Upon entering the tool’s website, users are directed to the homepage, which displays the tool’s name and a brief statement of its purpose. They then proceed to a participation consent page, confirming their voluntary participation.
2. Task instructions and optional user details: Before starting, users are presented with a short explanation of the fluency task. They have 2 min to generate as many animal names as possible. There is also an optional section for entering user details, though this is not required to proceed.
3. Completing the fluency task: Users are presented with a 2-min timer and a text box where they can type as many animal names as they can recall within the time limit. This task is designed to capture aspects of mental navigation and verbal fluency.
4. Viewing the results: After the task, users are taken to a results page, which displays a bar graph with their predicted scores across four dimensions:
   Creative thinking: the ability to generate creative solutions, based on the AUT (Acar & Runco, 2019)
  Creative potential: the propensity to act and achieve creative output across different domains, based on the ICAA (Diedrich et al., 2018)
  Intelligence: assessing fluid intelligence, based on various *Gf* tasks (Kenett et al., 2016)
  Openness to experience: assessing openness, based on the NEO-PI-3 (McCrae et al., 2005)

Each score ranges from 0 to 100, with 100 representing the highest value observed in our training sample, not an absolute or standardized benchmark.

5. Interpreting the scores: To avoid misinterpretation, and based on the approach taken by Pedersen et al. (2023), we emphasize that these scores are illustrative estimates rather than definitive measures of creativity, intelligence, or personality. The results are derived from our specific training dataset and serve as an approximation of broader cognitive abilities based on mental navigation metrics. Disclaimers are included to clarify that this tool does not function as a diagnostic assessment but rather as an exploratory approach to studying mental navigation.

## Results

We operationalize participants’ mental navigation based on their performance in the semantic fluency task and compute cognitive multiplex network features that quantify this process. We then examine how variability in these cognitive multiplex features predicts individual differences across our four dependent variables (creative potential, creative thinking, intelligence, and openness). Here, we report analysis only on the animal fluency task. However, a similar analysis of participants’ synonyms of hot conceptually replicated our findings for the animal task (see SI for full details of this analysis). We used LOOCV across the entire dataset to evaluate model generalizability. The *R*^2^ value was computed as the proportion of variance in each target variable explained by the model, while the reported *r* value reflects the Pearson correlation between the predicted and actual scores for each target across all LOOCV folds. This correlation indicates the consistency of the model's predictive performance.

**Fig. 3:**
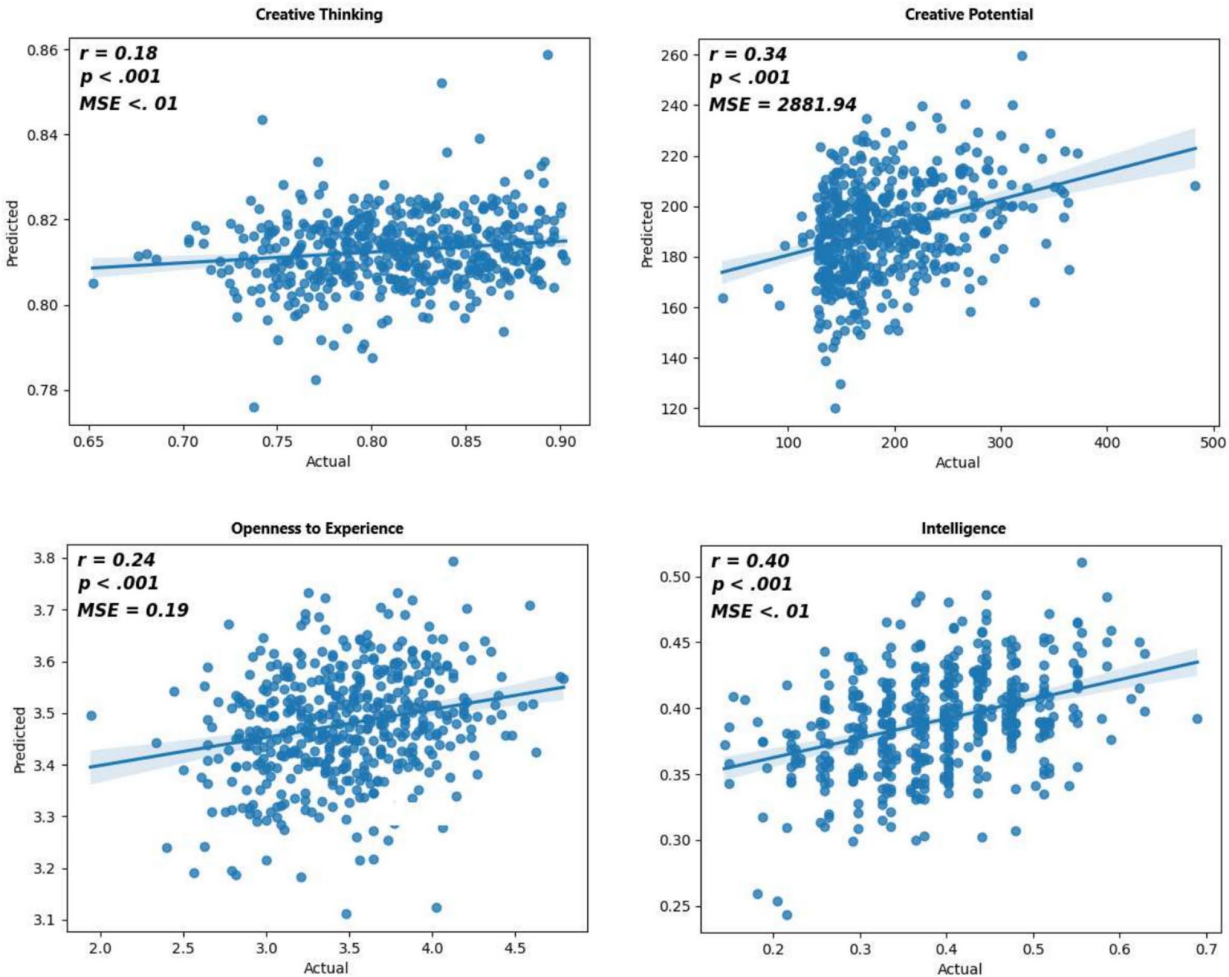
Scatter plots of correlations between predicted and actual scores of Creative Thinking (top left), Creative Potential (top right), Openness to Experience (bottom left), and Intelligence (bottom right). These plotscompare the actual values to the predicted values, providing a visual representation of the model's performance. The prediction ranges observed from the training data are as follows: creative thinking ranges from a minimum of .652 to a maximum of .903, creative potential ranges from 38 to 483, openness ranges from 1.9375 to 4.7916, and intelligence ranges from .1436 to .6897. These results highlight the model's capacity to effectively predict various cognitive capacities across a diverse set of data

*Creative thinking (AUT)*: The regression model significantly fit the data. The feature selection method chosen was correlation-based, *R*^2^ = .03, *F*(15,463) = 6.3, *p* < .001. The final model included fraction of responses in LVC, fraction of LVC accesses, start in LVC, accesses to LVC from animal, Norm1, Norm2, median permanence in LVC, coverage per response, max permanence in LVC, entropy of LVC accesses, fraction of incorrect spellings, distance from animal per response, median out, max out, and number of responses. The fraction of responses in LVC (*r* = − .11, *p* = .01) and fraction of LVC accesses (*r* = − .09, *p* = .04) were significant predictors of AUT. Finally, a comparison of our LVC model’s predicted AUT scores with participants’ true AUT scores revealed a significant positive correlation, *r*(477) = .18, *MSE* < .01, *p* < .001 (Fig. 3). Single-layer models did not provide statistically significant correlations (the highest correlation was for the synonym layer, *r*(477) = .06, *p* = .067).

*Creative potential (ICAA)*: The regression model significantly fit the data. The feature selection method chosen was CatBoost-based, *R*^2^ = .12, *F*(14,464) = 36.63, *p* < .001. The final model included number of responses, distance from animal per response, coverage per response, entropy of access to LVC, Norm2, fraction of LVC accesses, entropy of LVC responses, Norm1, accesses to LVC from animal, fraction of responses in LVC, entropy of LVC accesses, max out, median permanence in LVC, and max permanence in LVC. The number of responses (*r* = .27, *p* < .001), distance from animal per response (*r* = .12, *p* = .007), coverage per response (*r* = .11, *p* = .02), entropy of access to LVC (*r* = .15, *p* < .001), Norm2 (*r* = − .12, *p* = .007), fraction of LVC accesses (*r* = − .1, *p* = .03), entropy of LVC responses (*r* = − .16, *p* < .001), accesses to LVC from animal (*r* = − .09, *p* = .04), and fraction of responses in LVC (*r* = − .19, *p* < .001) were significant predictors of ICAA scores. Finally, comparison of our LVC model’s predicted ICAA scores with participants’ true ICAA scores revealed a significant positive correlation, *r*(477) = .34, *MSE* = 2,881, *p* < .001 (Fig. 3). All single-layer models provided statistically significant correlations, although weaker than the LVC model (all layers exhibited correlations close to .17, *p* < .001).

*Openness to experience*: The regression model significantly fit the data. The feature selection method chosen was correlation-based, *R*^2^ = .06, *F*(14,464) = 16.71, *p* < .001. The final model included the number of responses, fraction of responses in LVC, accesses to LVC from animal, Norm2, fraction of LVC accesses, entropy of access to LVC, Norm1, distance from animal per response, coverage per response, max permanence in LVC, median out, median permanence in LVC, entropy of LVC accesses, max out. number of responses (*r* = .18, *p* < .001), fraction of responses in LVC (*r* = − .14, *p* = .002), accesses to LVC from animal (*r* = − .1, *p* = .02) and Norm2 (*r* = − .09, *p* = .04) were significant predictors of Openness scores. Finally, comparison of our LVC model’s predicted openness scores with participants’ true openness scores revealed a significant positive correlation, *r*(477) = .24, *MSE* = .19, *p* < .001. (Fig. 3). Single-layer models did not provide statistically significant correlations (all layers exhibited correlations close to .05, *p* $\approx$ .11).

*Intelligence*: The regression model significantly fit the data, The feature selection method that was chosen was correlation-based, R^2^ = .16, F(15,463) = 67.25, *p* < .001. The final model included number of responses, coverage per response, entropy of access to LVC, Norm2, distance from animal per response, max permanence in LVC, Norm1, accesses to LVC from animal, start in LVC, max out, entropy of LVC accesses, fraction of LVC accesses, fraction of responses in LVC, median permanence in LVC, and fraction of incorrect spellings. The number of responses (*r* = .35, *p* < .001), coverage per response (*r* = .18, *p* < .001), entropy of access to LVC (*r* = .18, *p* < .001), Norm2 (*r* = − .16, *p* < .001), distance from animal per response (*r* = .15, *p* < .001), max permanence in LVC (*r* = .14, *p* = .002), Norm1 (*r* = − .12, *p* < .001), accesses to LVC from animal (*r* = − .1, *p* = .02), and start in LVC (*r* = .1, *p* = .03) were significant predictors of intelligence scores. Finally, a comparison of our LVC model’s predicted intelligence scores with participants’ true intelligence scores revealed a significant positive correlation, *r*(477) = .40, *MSE* < .01, *p* < .001 (Fig. 3). All single-layer models provided statistically significant correlations, although weaker than the LVC model (all layers exhibited correlations close to .23, *p* < .001).

## The High-level Cognition Predictor (HiCoP) tool

Finally, to disseminate our approach and demonstrate the potential of the cognitive smultiplex network model approach, we developed a proof-of-concept online tool based on our trained ML models for each of the four capacities we tested (creative thinking, creative potential, intelligence, and openness). Our tool sets the stage for developing models to predict high-level cognition in general based on mental navigation, and thus is called High-level Cognitive Prediction (HiCoP). HiCoP is a flexible, modular tool, that can be modified and extended for further applications related to quantifying mental navigation via the cognitive multiplex network approach. We focused on using the LVC model for HiCoP because in the above comparisons, the multiplex model based on the LVC systematically outperformed individual layers across the four tested constructs.

**Fig. 4:**
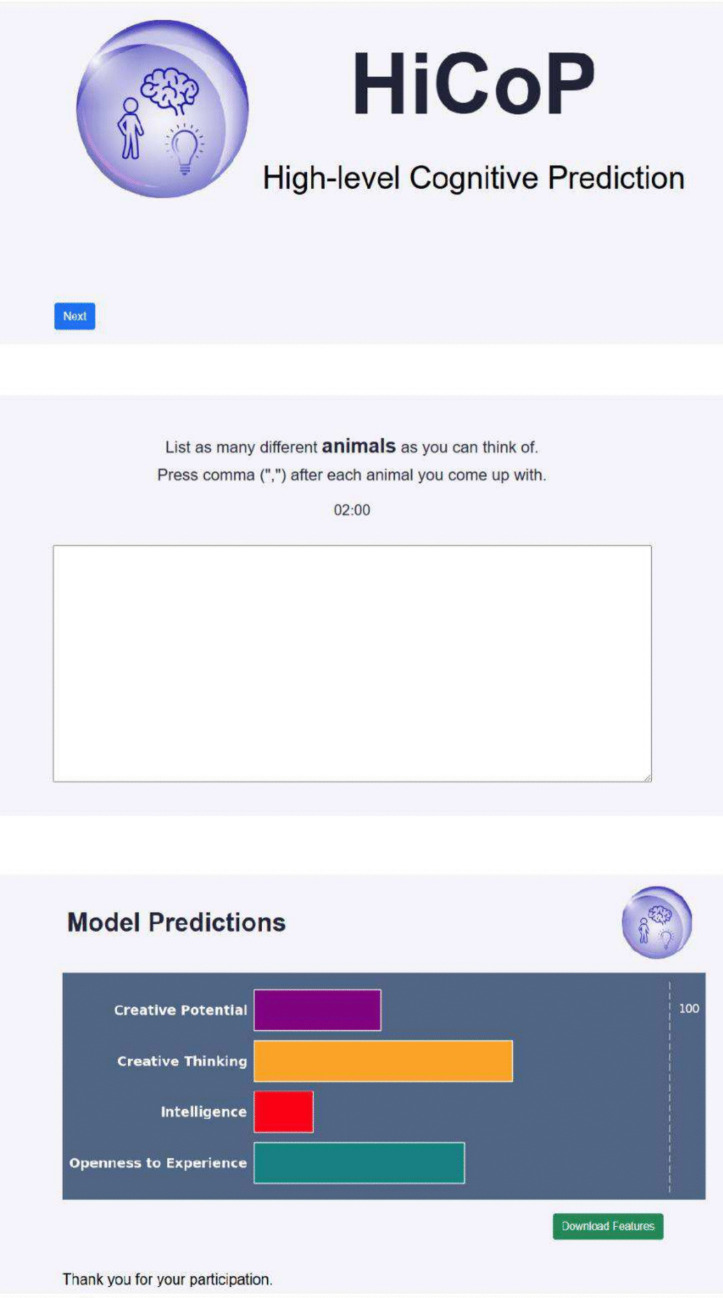
Illustration of HiCoP. Top: Landing page of HiCoP. Middle: The interface used for the animal fluency task. Bottom: A demonstration of predicted scores for the four cognitive capacities assessed by the model

The HiCoP tool, developed using oTree (Chen et al., 2016), provides users with an interactive platform to complete a fluency task by listing as many animals as they can within 2 min. In the background, the tool calculates multiplex features based on the user's input and applies a pre-trained ML model. Upon completion, the user receives output predicted scores for each of the four traits, displayed on a scale from 0 to 100 (Fig. 4). Based on Pedersen et al. (2023), these scores are represented as relative scores, and we clarify that these are simply predicted scores based on our models, to minimize any potential confusion among participants. HiCoP can be found at https://tinyurl.com/y4kszemt.

## Discussion

In the current study, mental navigation through memory (Benigni et al., 2021)— operationalized via a semantic fluency task—is examined for its ability to predict complex high-level cognitive behavior including creativity, intelligence, and openness to experience. To achieve this, the mental lexicon was modeled as a cognitive multiplex network incorporating both linguistic and conceptual information (Stella et al., 2024). Participants’ mental navigation performance over this network was then shown to correlate with individual differences in these measures and used to build ML models that accurately predict these scores. Thus, our work highlights the significance of using cognitive multiplex network modeling in empirical behavioral research, and further highlight its potential via an example proof-of-concept tool, HiCoP.

This work is based on recent applications of computational methods to study structure and processes in cognitive systems such as language and memory (Hills, 2025; Hills & Kenett, 2022; Siew et al., 2019). These advances have led to empirical investigation of the structure of memory as a graph, or network, and the processes operating over them, such as mental navigation (Benigni et al., 2021; Todd & Hills, 2020). However, these studies largely treat different linguistic levels—such as semantics and phonology—separately, and few studies have examined multidimensional cognitive systems, which encompass more than one type of information (Stella et al., 2024). These studies deal with analyzing a cognitive multiplex network, which comprises different layers, or networks, of information. Cognitive multiplex network research has demonstrated how such an approach can be uniquely used to study issues related to language, learning, development, creativity, personality, and clinical research (Castro, 2022; Castro & Stella, 2019; Ciaglia et al., 2023; Levy et al., 2021; Samuel et al., 2023; Stella, 2019; Stella & Kenett, 2019; Stella et al., 2017, 2018). For example, cognitive multiplex network modeling has been used to examine a general linguistic theory on lexical access and additional linguistic phenomena such as rhyming and priming (Levy et al., 2021; Stella, 2018), to study the personality trait openness to experience (Samuel et al., 2023), to examine different aspects of early language development (Ciaglia et al., 2023; Citraro et al., 2023; Stella et al., 2017, 2018), and to examine retrieval failures in clinical populations, currently focusing on people with aphasia (Baker et al., 2023; Castro & Stella, 2019; Castro et al., 2020; Stella, 2020). These examples highlight the general potential of applying cognitive multiplex network modeling in empirical research of typical and clinical populations across a wide range of cognitive domains (for a recent review, see Stella et al., 2024).

Several cognitive multiplex network studies have highlighted the role of a core in the multiplex network that cuts across all of the layers: the largest viable cluster (Stella & Kenett, 2019; Stella et al., 2018). This core, the LVC, is composed of highly general, frequent, and conceptually concrete words which are considered to facilitate language comprehension and processing. Importantly, the LVC emerges from the multiplexity of the mental lexicon and cannot be identified in single-layer modeling approaches. Stella and Kenett (2019) have shown that individuals with higher levels of creativity retrieve fewer words from the LVC and spend less time searching within it, in line with the idea that more creative individuals search more deeply and broadly through their memory (Kenett, 2023, 2025; Kenett & Faust, 2019b).

We examined the relation of the cognitive multiplex network measures—based on performance in the fluency tasks—and individual differences in creativity, intelligence, and openness measures. Across two different fluency tasks—listing animal names or synonyms of the word “hot”—we found that cognitive multiplex network measures quantifying the mental navigation during this task significantly predicted all of our dependent variables. Critically, our approach goes beyond standard and classical analysis methods of verbal fluency tasks, which focus on either the content produced or the number of responses produced by participants (Ardila et al., 2006; Henderson et al., 2023). However, the *R*^2^ across all models was quite modest, indicating that additional factors contribute to predicting such complex behaviors. Nevertheless, these results highlight, as a proof of concept, the role of mental navigation in such behaviors and how a cognitive multiplex network model can be used to capture this mental navigation.

Our analysis revealed multiple significant relations that further highlight the role of the LVC in complex behavior. Similar to the findings of Stella and Kenett (2019) and Samuel et al. (2023), the number of responses and coverage of responses were significantly related to openness and creativity. Thus, the current study replicates the findings of Stella and Kenett (2019), further highlighting the role of the LVC in complex behavior. Moreover, the current findings expand upon previous work (Samuel et al., 2023; Stella & Kenett, 2019), by moving from between-group comparisons to demonstrating how our approach can capture individual differences across various high-level cognitive capacities. Overall, as predicted, higher creativity and openness were related to relying less on the LVC and exploring more of the periphery of the mental lexicon.

Regarding the prediction of intelligence, we found opposite characteristics of mental navigation compared to creativity and openness: relying more on the LVC significantly predicted individual differences in intelligence, as measured via fluid intelligence (*Gf)*. These findings correspond to previous work showing that higher levels of *Gf* relate to a more structured mental lexicon (Kenett et al., 2016), likely enhancing efficient retrieval and manipulation of information. In contrast, a recent study found that higher crystallized knowledge, *Gc*, was related to a more flexible semantic memory structure (Li et al., 2024). Thus, while we did not assess *Gc* in the current study, it is likely that these two facets of intelligence differentially relate to reliance on the LVC.

Importantly, the LVC-based models showed systematically higher predictive power than their single-layer counterparts. This indicates that our findings emerge from the multiplex nature of the mental lexicon, which might exploit combinations of multidimensional associations to further sustain mental navigation, for example, combining both phonological similarities and synonyms to navigate associative knowledge (see also Levy et al., 2021, for a similar arguement). This quantitative comparison underlines the importance of investigating the mental lexicon from multiple perspectives at once (cf. Stella et al., 2024) to further extend psychological theories of mental navigation (for a review on the topic see also Hills, 2025).

More broadly, our findings further support the connection between the personality traits of openness to experience, creativity, and intelligence, and the mental lexicon. Traditionally, cognition and personality have been investigated separately, but a large body of work has linked creativity with openness, a personality trait that has been referred to as the “creativity trait” (Christensen, Cotter, et al., 2019; Christensen, Kenett, et al., 2018; Kaufman 2016; Oleynick 2017). Christensen, Kenett et al., (2018) have recently shown a relation between semantic memory structure and openness. The authors show how people that are more open had a more flexible, richly connected semantic memory network (Christensen, Kenett, et al., 2018). Our study provides further evidence supporting the relation between openness and the mental lexicon, drawing these two domains closer together. Thus, increased theoretical focus on the role of the mental lexicon in personality is needed (e.g., Dougherty & Guillette, 2018; Endler, 2000; Eysenck, 1993; Griffin et al., 2015). Recent studies have begun theoretically studying personality as a complex dynamic system using network science methodology (Beck & Jackson, 2021). Based on our current study, future cognitive multiplex network research should expand to incorporate a “personality” layer to more directly study how cognition and personality impact each other and interact to realize complex human behavior.

Our results provide further support that the mental lexicon can be modeled using a cognitive multiplex network approach, and that the characteristics of such a model are linked to complex cognitive traits such as language, development, and creativity in typical and clinical populations (Levy et al., 2021; Stella, 2019, 2020; Stella & Kenett, 2019; Stella et al., 2017; Stella et al., 2024). Furthermore, the link outlined here between the LVC and complex behavior indicates that multiplexity is an important feature of mental representations of linguistic knowledge. Since the LVC emerges from the multiplex interplay of semantic and phonological associations, the current work implies that cognitive multiplex networks represent a natural and convenient framework for exploring cognitive capacities, through quantitative and reproducible measurements, free from the constraints of subjective evaluations (e.g., self-report scales). Finally, our study proposes that one general cognitive mechanism of complex, high-level cognition is mental navigation. This potential general mechanism, and our ability to quantify and empirically assess it, advances theoretical and methodological research on these capacities.

From a more theoretical perspective, our work highlights the significance and general role of mental navigation in creativity, intelligence, and openness to experience. This indicates that at least these capacities share common cognitive functions, and therefore bias in one of these capacities should also be apparent in the others (see Herz et al., 2020, for a similar view). Specifically, our work corresponds with previous work by Hills and colleagues that argue for a general cognitive search mechanism that is governed by a central executive cognitive control mechanism (Hills, 2006; Hills et al., 2010; Todd & Hills, 2020). Indeed, cognitive control has been found to play a critical role in creativity, intelligence, and openness to experience at the cognitive and neural levels (Beaty, Chen, et al., 2018; Beaty et al., 2019; Benedek et al., 2014; Chen et al., 2019; Chrysikou, 2019; DeYoung et al., 2014; Krieger-Redwood et al., 2025). A recent study by Ovando-Tellez et al. examined the cognitive and neural correlates of semantic memory search related to creativity (Ovando-Tellez, Benedek, et al., 2022). The authors show how semantic memory search is primarily related to executive processes, but also to semantic memory structure. Thus, the authors provide empirical evidence of how cognitive search is an active, goal-directed process in line with the general central executive model of cognitive search (Hills et al., 2010). However, mental navigation—or cognitive search—is only one mechanism that drives high-level cognition. The structure of one’s mental lexicon has been shown to constrain such a search process operating over it (Benedek et al., 2023; Hills & Kenett, 2025; Johnson & Hass, 2022; Kenett, 2025; Wulff et al., 2020), which is in accord with classical cognitive theory (Collins & Loftus, 1975). Therefore, the cognitive multiplex network model provides a powerful and general approach to empirically study both aspects of how the structure (e.g., LVC) and processes that operate over it (e.g., mental navigation) relate generally to high-level cognition (cf. Hills & Kenett, 2022).

## Limitations and future directions

A few limitations to our study should be mentioned. First, prediction of creative thinking was the least successful compared to the other three examined variables. As ML models learn which features and measures to use in prediction based on the variance between participants, it might not be surprising that the models were not able to provide very good prediction for creative thinking scores. Thus, this low success in predicting creative thinking is likely due to the narrower distribution of its measurement. Overall, the predicted *R*^2^ of all models was rather modest, ranging from 0.18 to 0.40. This could be related to sample size, despite the large sample, or to assessment issues of the dependent variables. Furthermore, high-level cognition involves multiple cognitive capacities (e.g., Benedek & Fink, 2019; Benedek et al., 2023), so it is not surprising that our model captures only a fraction of the variance. Nevertheless, our ability to predict such cognitive capacities based solely on a 2-min verbal fluency task indicates the strength and feasibility of our approach. Further research is needed to further develop and strengthen the feasibility of our approach, based on larger samples and additional validation studies.

Another potential limitation concerns the generalizability of our results: Can mental navigation operating over the mental lexicon predict general cognitive capacities that are not specific to the animal category? First, the animal category is the most typically used semantic fluency category in clinical and typical research (Ardila et al., 2006; Troyer et al., 1998). Despite some criticism about using this specific category to study mental navigation (Ovando-Tellez, Benedek et al., 2022), it is still widely accepted as the gold standard in studying such cognitive processes (Benigni et al., 2021; Siew et al., 2019). In addition, we examined the success of our approach using two very different categories—animals and synonyms of hot. The replication of our results based on the animal category with that of the hot synonyms (see SI) strengthens its generality. However, further research is needed to examine whether other types of fluency tasks (e.g., Unsworth et al., 2014) can be operationalized on our cognitive multiplex network model and be used to similarly predict high-level cognitive capacities.

Additionally, we acknowledge the limitations of our cross-validation method. Given our relatively small dataset and the need to ensure methodological consistency with prior work (e.g., Samuel et al., 2023; Stella & Kenett, 2019), we opted for leave-one-out cross-validation (LOOCV), as it maximizes training data and enables direct comparability with previous studies (Cheng et al., 2017; Roberts et al., 2017; Yates et al., 2023). LOOCV ensures that each data point is tested exactly once, making full use of the dataset while minimizing unused information. This method also allows for a direct one-to-one comparison of prediction errors across individual participants, reducing variance in the estimates. While other validation methods are often preferred in larger datasets, LOOCV has been shown to be the method of choice for small datasets such as ours. Specifically, Roberts et al. (2017), in a thorough review of LOOCV, argue that it is the preferred method when computationally feasible, particularly for small datasets. This is because of the reduction in bias as the size of the training set increases, such that *k* = *n* (i.e., leave-one-out) has the lowest bias of all *k* values in *k*-fold cross-validation (Roberts et al., 2017). Future studies are needed to collect even larger datasets, making it possible to examine various *k*-fold methods to assess the generalizability of our approach and mitigate potential overfitting concerns.

Finally, future research is needed to examine our approach in predicting other complex cognitive traits. Showing evidence of a relation between the mental lexicon and other cognitive traits would broaden the scope of findings shown in our study and widen the variety of objective methods to study cognition. Moreover, using a shorter semantic fluency task, of 30 s for example, in order to compute multiplex network measures could also be an interesting step forward, because it would make this method even more practical and applicative in ecological settings. Finally, the reliability of our approach should be tested in relation to potential manipulations or interventions in the semantic fluency task. For example, examining how the typical instruction manipulation in creativity research to “be creative” impact such mental navigation (Wei et al., 2024); or how this approach be used for diagnostic purposes in clinical research of populations that suffer from thought disorders (Kenett & Faust, 2019a), similar to pioneering research by Castro et al. (2022).

## Conclusions

In summary, the results of the current study demonstrate that it is possible to predict complex cognition using a cognitive multiplex network model and a very short task, even when data size is limited, which improves when data size is more suitable for ML tasks. Thus, this work demonstrates how computational tools can be used in empirical research to investigate and predict complex cognitive capacities, specifically mental navigation. Furthermore, the study demonstrates the ability to predict complex behavior from simple behavioral tasks such as semantic fluency, an approach that goes beyond standard methods to analyze such data—methods that have shown poor predictive ability. Theoretically, our findings highlight the general role of mental navigation in high-level cognition, and further draw personality and cognition closer together. In addition, our results provide initial evidence for the ability to develop automatic, objective scoring of openness, creativity, and intelligence. Such quantitative direction has significantly advanced creativity research over the past decade (Beaty & Johnson, 2021; Dumas et al., 2021; Organisciak et al., 2023).

Our work primarily answers a recent call for more computational modeling in creativity research (Lloyd-Cox et al., 2023), but extends more broadly to high-level cognition and personality research. Given the significance of high-level cognition, it is critical that we push forward in studying these processes at the cognitive and neural levels. Moreover, identifying common mechanisms across these high-level cognitive processes is critical to their elucidation. As we show here, one such mechanism is mental navigation.

## Supplementary Information

Below is the link to the electronic supplementary material.

Supplementary file1 (DOCX 206 KB)

## Data Availability

Data and code used in the manuscript are available on the Open Science Framework at https://osf.io/6axuq/.

## Code availability

Data and code used in the manuscript are available on the Open Science Framework at https://osf.io/6axuq/.
