# Supplementary material for: A cognitive multiplex network approach to investigate mental navigation and predict high-level cognition
Source: Behav Res Methods. 2025 Aug 25;57(10):268. doi: 10.3758/s13428-025-02748-6 (PMC12378698; doi:10.3758/s13428-025-02748-6)
Supplement: Supplementary file 1 — Supplementary file1 (DOCX 206 KB) [file 13428_2025_2748_MOESM1_ESM.docx]

# **Mental navigation predicts higher-level cognition**

**Supplementary Information**

**Analysis based on the synonyms to Hot category**

We conduct a similar analysis with the synonyms to Hot fluency task as an operationlization of participants’ mental navigation in a different category (than animals). Similarly to the animal category, we investigated the relationship between various cognitive multiplex network properties, including reliance on the Largest Viable Cluster (LVC), and individual differences in Openness, creative potential, creative thinking, and intelligence levels using Pearson’s correlation analysis. For the prediction model, we employed a linear regression approach. The features used in the regression model were selected through two different feature selection methods: correlation-based and CATBoost. These methods rank features from most to least important, allowing us to test several models by starting with the top-ranked feature and iteratively adding those of lesser significance. The retained features are essential for accurately predicting the dependent variable. The analysis below focuses exclusively on the animal fluency task.

*Creative Thinking*: The regression model significantly fit the data, the feature selection method that was chosen was Correlation-based, R^2^ = .04, F(15,463) = 7.25, *p* < .001. The final model included: Norm1, Fraction of Responses in LVC, Start in LVC, Coverage per Response, Median Permanence in LVC, Max Out, Fraction of LVC Accesses, Max Permanence in LVC, Accesses to LVC from Hot, Distance from Hot per Response, Entropy of LVC Accesses, Entropy of Access to LVC, Fraction of Incorrect Spellings, Number of Responses and Median Out. Norm1 (r = .12, *p* = .007) Fraction of Responses in LVC (r = -.1, *p* = .03) were significant predictors of creative thinking. Finally, comparing our LVC model’s predicted AUT scores with participant’s true creative thinking scores revealed a significant positive correlation, r(477) = .20, MSE < .01, *p* < .001 (**Figure SI 1**). Single-layer models did not display any predictive power for creative thinking (correlations *r* between .03 and .05, *p* > .106).

*Creative Potential*: The regression model significantly fit the data, the feature selection method that was chosen was Correlation-based, R^2^ = .07, F(14,464) = 12.31, *p* < .001. The final model included Number of Responses, Fraction of Incorrect Spellings, Max Out, Norm2, Median Out, Median Permanence in LVC, Entropy of LVC Responses, Coverage per Response, Entropy of LVC Accesses, Entropy of Access to LVC, Accesses to LVC from Hot, Max Permanence in LVC, Fraction of LVC Accesses and Distance from Hot per Response. Number of Responses (r= .16, p = .0005) was a significant predictor of creative potential scores. Finally, comparing our LVC model’s predicted creative potential scores with participant’s true creative potential scores revealed a significant positive correlation, r(477) = .26, MSE = 3032, *p* < .001 (**Figure SI 1**). At the selected significance level, predictions coming from single-layer models did not correlate with creative potential (correlations *r* between .01 and .06, *p* > .071). This is different from what happened with the animal category. In the main text, single-layer models displayed some predictive power for creative potential, although weaker than what the LVC model displayed.

*Openness*: The regression model significantly fit the data, the feature selection method that was chosen was Correlation-based, R^2^ = .04, F(15,463) = 2.62, *p* < .001. The final model included Fraction of LVC Accesses, Fraction of Responses in LVC, Number of Responses, Coverage per Response, Entropy of LVC Accesses, Norm1, Distance from Hot per Response, Accesses to LVC from Hot, Entropy of Access to LVC, Max Permanence in LVC, Median Out, Median Permanence in LVC, Max Out, Start in LVC and Fraction of Incorrect Spellings. Finally, comparing our model’s predicted Openness scores with participant’s true Openness scores revealed a significant positive correlation, r(477) = .19, MSE = .19, *p* < .001 (**Figure SI 1**). Single-layer models did not display any statistically significant correlation with openness levels (correlations *r* between .01 and .03, *p* > .35).

*Intelligence*: The regression model significantly fit the data, the feature selection method that was chosen was CATboost-based, R^2^ = .05, F(14,464) = 8.0, *p* < .001. The final model included Coverage per Response, Number of Responses, Distance from Hot per Response, Entropy of Access to LVC, Accesses to LVC from Hot, Norm1, Fraction of LVC Accesses, Norm2, Median Permanence in LVC, Start in LVC, Entropy of LVC Accesses, Entropy of LVC Responses, Fraction of Responses in LVC and Median Out. Coverage per Response (r= .13, p = .005), Number of Responses (r = .09, *p* = .04), Start in LVC (r = .1, *p* = .03) were significant predictors of intelligence scores. Finally, comparing our model’s predicted intelligence scores with participant’s true intelligence scores revealed a significant positive correlation, r(477) = .22, MSE < .01, *p* < .001 (**Figure SI 1**). Single-layer models did not display any statistically significant correlation with intelligence scores (correlations *r* between .02 and .05, *p* > .12).

**SI Figure 1.**

*Scatter plots of correlations between predicted and actual scores of Openness (top left), Creative thinking (top right), Creative potential (bottom left), and Intelligence (bottom right)*


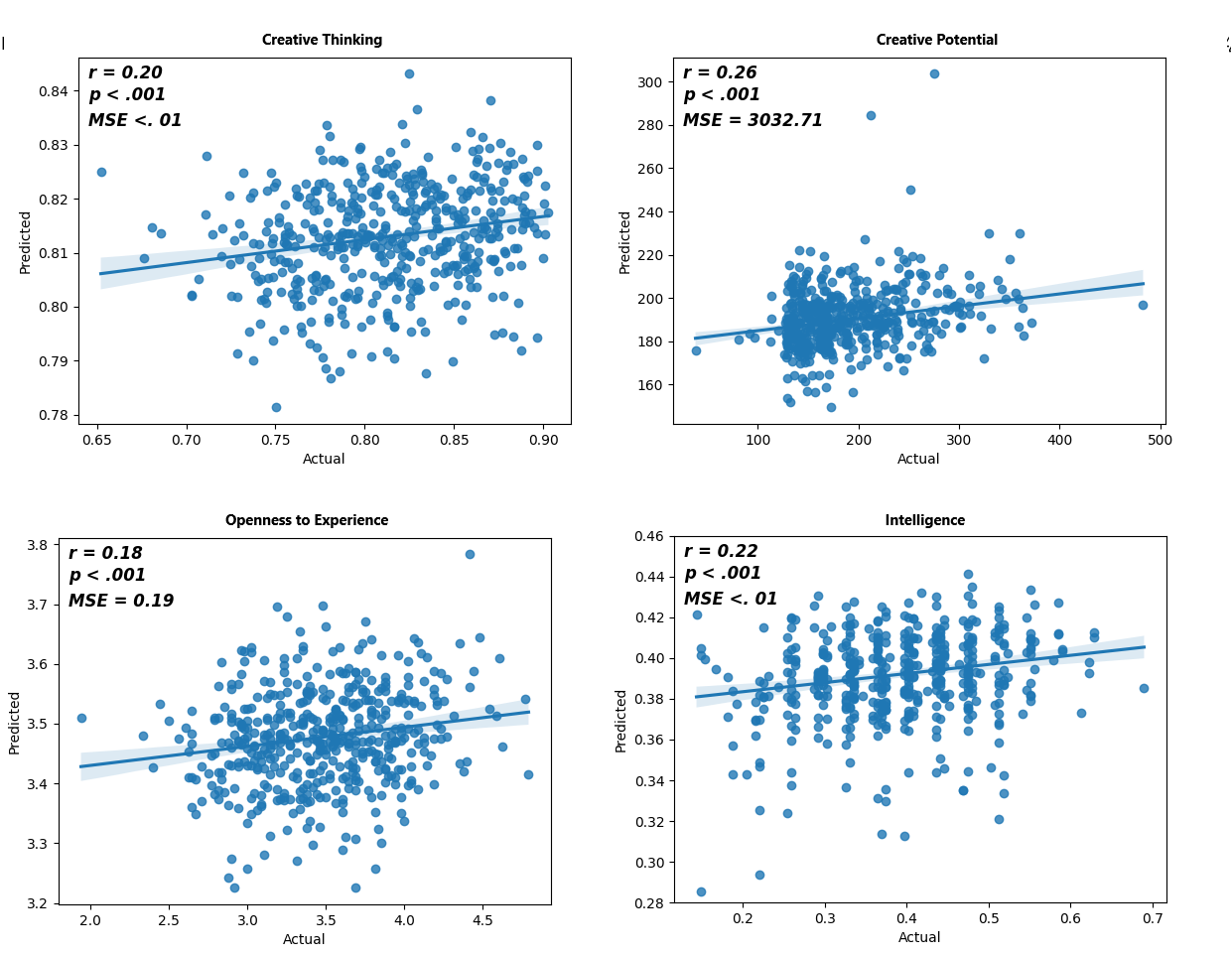


*Note* – These plots compare the actual values to the predicted values, providing a clear visual representation of the model's performance. The prediction ranges observed from the training data are as follows: creative thinking ranges from a minimum of 0.652 to a maximum of 0.903; creative potential ranges from 38 to 483; openness ranges from 1.9375 to 4.7916; and intelligence ranges from 0.1436 to 0.6897. These results highlight the model's capacity to effectively predict various cognitive traits across a diverse set of data.
